# Rethinking the “transition phase” in ARDS: can we afford to overlook the physiology behind NMBA weaning and PSV failure??

**DOI:** 10.1186/s13613-025-01565-6

**Published:** 2025-09-30

**Authors:** Nan Xiong, Yinde Huang

**Affiliations:** 1 Department of Gastroenterological Surgery, Peking University People’s Hospital, Beijing, 100044 China; 2 Department of Breast and Thyroid Surgery, Chongqing General Hospital, Chongqing, 401147 China

**To the editor**

We read with great interest the recent observational study by Haudebourg et al. on the transition phase in moderate-to-severe acute respiratory distress syndrome (ARDS), which highlights the high failure rates associated with neuromuscular blocking agent (NMBA) withdrawal and pressure support ventilation (PSV) initiation during this understudied interval [1]. While the authors’ efforts to define and characterize this “gray zone” are commendable, we believe two critical gaps—linked to both physiology and monitoring—deserve further scrutiny.

First, the reported 38% failure rate of NMBA withdrawal within 48 h is concerning not only due to its association with increased 28-day mortality (31% vs. 12%, *P* = .003), but also because it underscores the lack of operationalized physiological thresholds to guide NMBA discontinuation [1]. The authors identified low pH, low PaO₂/FiO₂, and extreme driving pressures as independent predictors. Yet these metrics, though statistically significant, demonstrated limited predictive accuracy (AUCs of 0.58–0.63) [1]. Should we be comfortable making clinical decisions in such a physiologically vulnerable phase using tools with such poor discriminative power?

Moreover, the non-linear relationship between driving pressure and NMBA failure—where both high and low values (< 12 or ≥ 14 cmH₂O) conferred increased risk—raises an important mechanistic dilemma. Prior literature supports the prognostic role of driving pressure in ARDS mortality [2]; however, this study suggests that overly low driving pressures may indicate an inappropriately low tidal volume for a given respiratory system compliance, leading to “air hunger” and patient–ventilator asynchrony. Could it be that our overzealous commitment to low tidal volume ventilation inadvertently primes patients for NMBA reinitiation due to P-SILI (patient self-inflicted lung injury)? [3].

Second, the PSV failure rate of 57% is startling—particularly when coupled with the finding that 43% of patients exhibited tidal volumes exceeding 8 mL/kg predicted body weight (PBW) during PSV [1]. Despite moderate inspiratory support and seemingly acceptable gas exchange, tidal volume emerged as the only independent predictor of PSV failure (OR, 1.28; 95% CI, 1.06–1.56), with a threshold of > 9.3 mL/kg PBW demonstrating 91% specificity for predicting failure [1]. These findings resonate with prior work in noninvasive ventilation, where high tidal volumes (> 9.5 mL/kg PBW) predicted both NIV failure and mortality in de novo hypoxemic respiratory failure [4]. Yet, no direct measures of respiratory effort (e.g., esophageal pressure swings, P0.1, EAdi) were available. Without this information, how can we differentiate whether high tidal volumes reflect improving respiratory mechanics—or, more alarmingly, excessive respiratory drive leading to P-SILI?

These two findings converge on a central issue: the lack of physiologic granularity during the ARDS transition phase. Current practice focuses heavily on binary milestones—e.g., stop NMBA or switch to PSV—while ignoring the complex, dynamic interplay between lung mechanics, respiratory drive, and patient effort. In an era where the concept of “lung-protective weaning” is gaining traction [5]how can we continue to ignore effort-sensitive metrics when they may hold the key to safer transitions?

In conclusion, we urge future studies to integrate real-time respiratory drive monitoring and to consider individualized thresholds for NMBA withdrawal and PSV initiation. Until then, we risk managing the most physiologically unstable phase of ARDS with dangerously blunt tools.

## Declarations

### Use of AI-assisted editing

This manuscript was edited for grammar, clarity, and style using ChatGPT (OpenAI), a large language model–based assistant. The tool was employed solely for language refinement after the scientific content had been finalized. No AI-generated content was used to develop the study design, analyze data, or draw scientific conclusions.

## References

[1] Haudebourg AF, et al. Factors influencing the transition phase in acute respiratory distress syndrome: an observational cohort study. Ann Intensive Care. 2025;15:71. 40415127 10.1186/s13613-025-01484-6 PMC12104120

[2] Amato MB, et al. Driving pressure and survival in the acute respiratory distress syndrome. N Engl J Med. 2015;372:747–55. 25693014 10.1056/NEJMsa1410639

[3] Yoshida T, et al. Volume-controlled ventilation does not prevent injurious inflation during spontaneous effort. Am J Respir Crit Care Med. 2017;196:590–601. 28212050 10.1164/rccm.201610-1972OC

[4] Carteaux G, et al. Failure of noninvasive ventilation for de Novo acute hypoxemic respiratory failure: role of tidal volume. Crit Care Med. 2016;44:282–90. 26584191 10.1097/CCM.0000000000001379

[5] Goligher EC, et al. Effect of Lowering Vt on mortality in acute respiratory distress syndrome varies with respiratory system elastance. Am J Respir Crit Care Med. 2021;203:1378–85. 33439781 10.1164/rccm.202009-3536OC

